# Endometrial Stromal Cells from Endometriosis Patients Reflect Lesion-Type-Specific Heterogeneity

**DOI:** 10.3390/cells14231891

**Published:** 2025-11-28

**Authors:** Daniel Rodriguez Gutierrez, Marianne R. Spalinger, Alina Astourian, Olivera Evrova, Lucie Berclaz, Monique Hartmann, Ioannis Dedes, Patrick Imesch, Julian M. Metzler, Isabelle Witzel, Mohaned Shilaih, Valentina Vongrad, Brigitte Leeners

**Affiliations:** 1 Department of Reproductive Endocrinology, University Hospital Zurich, 8091 Zurich, Switzerland; 2 Department of Gynecology, University Hospital Zurich, 8091 Zurich, Switzerland

**Keywords:** endometriosis, endometrium, endometriotic lesion types, endometrioma, deep infiltrating endometriosis, stromal cells, cell isolation, primary cell model

## Abstract

**Highlights:**

**What are the main findings?**

- Endometrial stromal cells from endometriotic lesions recapitulate the disease heterogeneity of endometriosis.
- Endometrial stromal cells show lesion-specific differences in migration, proliferation, contractility, and extracellular matrix.

**What is the implication of the main finding?**

- Endometrial stromal cells represent an excellent tool to study patho-mechanisms involved in endometriosis.
- Endometrial stromal cells represent a novel model to test therapeutic approaches that consider disease complexity and heterogeneity.

**Abstract:**

Endometriosis, a disease affecting about one out of ten women, is characterized by the growth of endometrial-like tissue outside the uterine cavity. There is significant disease heterogeneity, but the pathophysiological mechanisms underlying differences in clinical presentation are poorly understood. Here, we investigated endometrial stromal cells (ESCs) from different types of endometrial lesions (endometrioma, superficial, and deep endometrial lesions), which revealed distinct differences in proliferation, migration, and contractility among different lesion types and when compared to ESC from normal (eutopic) endometrium. In particular, ESCs from endometriotic lesions showed reduced proliferation but increased migratory capacity, an effect most pronounced in endometrioma ESCs but also evident in ESCs from superficial and deep lesions. ESCs from superficial and deep lesions—but not those from endometrioma—showed increased contractility, a feature involved in tissue scarring and pain perception. Transcriptomics and proteomics revealed changes in genes and proteins involved in cell division, proliferation, extracellular matrix organization, and migration in endometriosis vs. healthy ESCs. Overall, our results demonstrate that stromal cells from different endometriotic lesions show distinct in vitro phenotypes that might explain differences in clinical presentation. Further, these cells represent an excellent in vitro model for studying patho-mechanisms involved in endometriosis heterogeneity.

## 1. Introduction

Endometriosis (EM) is a chronic gynecological disorder characterized by the presence of endometrium-like tissue outside of the uterine cavity [1]. Worldwide, EM affects 5–10% of women in reproductive age. Symptoms include chronic pelvic pain, general ill-being, and infertility, and impose a significant burden on affected patients [2]. In the past decades, research on endometriosis has increased, but the mechanisms involved in lesion initiation and development are still not fully understood, and a better understanding of disease-driving mechanisms is urgently needed to develop improved, and ideally patient-and/or lesion-specific treatment options.

As one of the leading theories on EM development, Sampson’s theory postulates that EM is linked to retrograde menstruation [3]. During retrograde menstruation, clusters of shed endometrial stromal cells (ESCs) and endometrial epithelial cells (EECs) reach the peritoneum through the fallopian tubes. EM develops when these cells are capable to attach to pelvic and intra-abdominal surfaces, and manage to successfully implant and proliferate [4,5]. However, especially in deep infiltrating lesions, endometriosis is considered to develop locally from pluripotent stem cells [6,7]. While EM lesions can occur in extra-pelvic sites, they are mostly found in the peritoneum and organs in the pelvis [8]. Among these, the most common types are superficial (peritoneal) lesions deep infiltrating lesions, and ovarian endometriotic cysts (endometriomas) [9]. Traditionally, researchers viewed EM as a single, uniform disease, but extensive heterogeneity has been observed in endometriotic lesions even among lesions from the same patient [10]. The clonal origin [11] and the ~50% hereditability [12] are additional evidence of a highly heterogeneous and complex disease.

ESCs play a central role in EM development [13,14] as their adhesive properties allow attachment to and invasion of ectopic sites [15]. ESCs secrete pro-inflammatory factors, chemokines, and cytokines [16]; promote angiogenesis [17]; and show altered hormonal crosstalk that promotes survival and growth of ectopic endometrial tissue [18]. Given their importance for EM development, studying ESCs has the potential to unravel lesion-specific disease mechanisms and to develop and test new treatment approaches. However, while immortalized cell lines do not represent patient- or lesion-specific differences, obtaining primary cells from patient material can be difficult, and isolation processes might alter cellular functions [19,20,21,22]. Thus, primary cells that retain individual and lesion-specific characteristics during and after isolation are required to better understand disease mechanisms and to develop more specific, and ideally personalized, treatment options.

The aim of this study was to isolate stromal cells from eutopic endometrium and different EM lesions to assess potential lesion-specific differences. Our study revealed crucial differences in key features of ESCs, such as their proliferative and migratory potential (assessed with a gap covering assay), their mRNA and protein expression (assessed via RNA sequencing and proteomics), and their contractibility (assessed in a specialized cell culture system). These differences were not only observed between ESCs from eutopic endometrium and those obtained from EM lesions, but also among ESCs obtained from different EM lesion types. Notably, ESCs showed lesion-type-specific differences linked to typical clinical features of the lesion type from which the ESCs originated. Thus, primary ESCs might present an ideal model to investigate disease mechanisms and to identify new (lesion-specific) targets for endometriosis therapy.

## 2. Materials and Methods

***Tissue collection.*** Eutopic and ectopic endometrial tissue from endometrioma, superficial, and deep infiltrating lesions was collected from women undergoing surgery due to EM. Biopsies were resected by experienced surgeons in accordance with local ethics policies and guidelines (BASEC #2020-02117). Signed informed consent was obtained from each patient prior to inclusion in the study. Patients were tested negative for HBsAg, HCV, HIV, and syphilis prior to experiments. The exact number of samples for each lesion subtype that was used for each analysis is given in the respective figure legends. In total, endometrial stromal cells from 24 biopsies and a total of 18 patients were used for this study. Data regarding cycle day and hormonal treatment of the patients is given in Supplementary Table S1.

***Endometrial stromal cell isolation.***

Enzymatic digestion-based isolation: Tissue pieces were dissociated using a gentleMACS™ Octo Dissociator (Miltenyi Biotec, Bergisch Gladbach, Germany) in dissociation medium containing 1 mg/mL collagenase I (Thermo Fisher, Waltham, MA, USA, #17100017), 50 ug/mL DNase I (Roche, Basel, Switzerland, #7002221), and 10 uM ROCK inhibitor (Sigma-Aldrich, St. Louis, MO, USA, #SCM075) for 1 h at 37 °C and passed through 70 um cell strainers. The flow-through fraction containing ESCs was washed with PBS (Gibco, Waltham, MA, USA, 10010-015) and seeded in ESC culture medium (DMEM/F12, Sigma, St. Louis, MO, USA, D6434; 10% FBS, Himedia, Mumbai, India, #10432; 1% antibiotic/antimycotic solution, Thermo Fisher Scientific, #15240062). Fractions that were retained in the filter (mainly EECs) were resuspended in EEC medium (Gibco, #10785-012) and seeded on Matrigel (Merck, Darmstadt, Germany) coated 24-well plates.

Tissue outgrowing/migration-based isolation: 1.5 mm^2^ tissue pieces were washed with PBS, placed in 10 cm tissue culture plates, and allowed to adhere for 10–15 min at room temperature before ESC culture medium was added. The cultures were left untouched for 5 days, after which the media was changed every two days. Once colonies reached 80% confluency, the cells were washed with PBS and detached using trypsin-EDTA (Sigma-Aldrich, #T2601) and transferred into culture flasks for further expansion. Cells were passaged when reaching a confluency of 80%.

Phase contrast images were taken every second day from the time of tissue processing (day 0) to the first passage (days 11–20). At passage (P)1, ESCs were characterized by immunofluorescence (IF) and flow cytometry for the expression of EEC and ESC markers. Cells from P1–P3 were used for all other experiments.

***Immunofluorescence***. For immunofluorescence, ESCs from P1 were seeded in Ibidi chamber slides (Ibidi, Gräfelfing, Germany, #80826-IBI) and left to adhere overnight. The cells were washed twice with PBS and fixed for 15 min with pre-cooled acetone at −20 °C; washed 3 times with PBS; unspecific binding sites blocked with 3% BSA in PBS for 1 h at room temperature; and incubated overnight at 4 °C with primary antibodies diluted in 1%BSA containing PBS. On the next day, the slides were washed 3 times in PBS; incubated with secondary antibodies and AlexaFluor647-labelled phalloidin (Sigma #A22287, 1:500) for one hour; mounted with DAPI containing mounting solution (Thermo Fisher, #62248); and images taken with an LAX confocal microscope. Antibodies and dilutions are given in Supplementary Methods. Image composites were created using FIJI software (Version 2.9.0) [23].

***Flow cytometry.*** ESCs from P1 were washed twice in FACS Buffer (PBS + 2% FBS + 0.1% NaN3) and incubated for 15 min with BD human Fc block (BioLegend, San Diego, CA, USA, 1:100) prior to surface staining for 40 min at 4 °C. The cells were washed with FACS buffer and resuspended in 200 μL PBS supplemented with 2 mM EDTA (Applichem, Darmstadt, Germany, #A4892). For intracellular staining, the cells were fixed for 40 min in 4%PFA (EMS Chemie, Domat/Ems, Switzerland, #15712) at 4 °C, permeabilized in 1% Tween in PBS for 10 min and stained with antibodies against ERα and ERβ. Samples were washed, resuspended in FACS buffer, and measured on a BD Celesta™ [24]. The gating strategy and gate settings can be found in Supplementary Figure S1. Antibodies and dilutions are given in Supplementary Methods.

***Proliferation and migration assay.*** ESCs (P1–P3) were seeded in silicon inserts (Ibidi, #80209) and grown to confluency before removing the inserts to create a standardized gap. Cells were then cultured either with DMEM/F12 supplemented with 10% FCS (normal growth medium) or with reduced nutrient medium (DMEM/F12 supplemented with 1% FCS). Images of the gap were taken every 30 min with a live imaging microscope from Olympus (IX81) (Olympus Corporation, Tokyo, Japan). FIJI/ImageJ Software (Release/Verion 2.9.0) was used to measure the gap area in each image. Experiments were performed in triplicate.

***Contractility Assay.*** The contractility assays were performed in 4Dcell SmartHeart Plates from 4DCell according to the manufacturer’s instructions and as described in Supplementary Methods.

***Proteomics and RNA Sequencing.*** For proteomics analyses, ESCs (P1–P3) were lysed in M-PER (Thermo Fisher Scientific, #78505), incubated for 15 min on ice, and the lysates were centrifuged for 10 min at 10,000× *g* at 4 °C. The supernatant containing proteins was stored at 20 °C until sending to the Functional Genomic Center Zürich (FGCZ) for standard proteomics analysis. For RNA sequencing, RNA was isolated using the Qiagen RNAesy mini kit (Qiagen, Venlo, The Netherlands, #74104) according to the manufacturer’s instructions. RNA was stored at −80 °C until transcriptomic analysis at the FGCZ. Details on proteomics and RNA sequencing methods/data analysis are given in Supplementary Methods and included previously described methodology [25,26,27,28,29,30,31,32,33]. Proteomics data have been deposited into the PRIDE (http://www.ebi.ac.uk/pride (accessed on 29 July 2025)) repository (PXD066685); RNA sequencing data have been deposited in NCBI’s gene expression omnibus (GEO; GSE303635).

## 3. Results

### 3.1. Outgrowing of Cells from Endometriotic Lesions Yields Pure Fibroblast-like Stromal Cells

**Figure 1:**
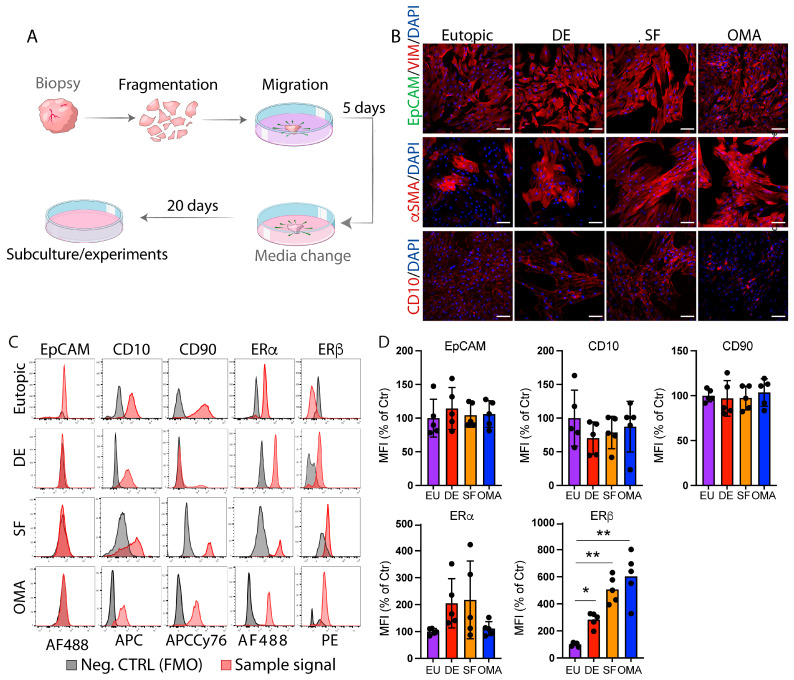
Outgrowing of cells from tissue pieces yields highly pure ESCs. (**A**) Schematic overview of the cell isolation method via migration/growing out from tissue pieces. (**B**) Representative images of ESCs that were grown out from the indicated lesion types and stained for EpCAM (green) and Vimentin (red; top panel); α smooth muscle actin (αSMA, middle panel, red); or CD10 (red, bottom panel). Nuclei were stained with DAPI (scale bar: 50 µm). (**C**) Flow cytometry analysis using the epithelial cell marker EpCAM and the stromal cell markers CD90 and CD10. Expression in/on the cells (red curves) were compared to respective FMO controls (gray curves). FMO (fluorescence-minus-one): samples that were fully stained except for the one marker/color that is shown on the *x*-axis of the respective plots. (**D**) Statistical analysis of flow cytometry analysis. * *p* < 0.05, ** *p* < 0.001; MFI: mean fluorescent intensity. For each lesion type, n = 5.

As stromal cells, and especially fibroblasts, are heavily influenced by mechanical cues and might undergo significant changes in their behavior depending on the isolation technique [34], we tested two protocols of stromal cell isolation: in a first approach to isolate and characterize endometrial stromal cells (ESCs) from different endometriosis lesions, we applied a commonly used method consisting of tissue digestion followed by filtration [35]. However, this technique yielded mixed cellular fractions that contained ESC and endometrial epithelial cells (EECs, Supplementary Figure S2), and cell yield was poor, especially in tissues from highly fibrotic EM lesions (Supplementary Figure S2). Thus, we next tested a tissue outgrowing/migration-based method to isolate untouched stromal cells. Tissue pieces were placed on cell culture plates and cells were left to grow out for several days (Figure 1A). This method yielded highly sufficient numbers of pure stromal cells that resembled fibroblasts (elongated, spindle-like shape, round nuclei [36]; Figure 1B) and was subsequently used for all experiments and analyses.

Immunofluorescence and flow cytometry confirmed the stromal cell nature of these cells (negative for epithelial cell adhesion molecule (EpCAM), positive for the stromal cell markers CD10 and CD90 [37,38]; Figure 1B,C). ESCs from all three lesion types (superficial and deep (DE) lesions, endometrioma) showed increased levels of estrogen receptor (ER)-β when compared to ESC from eutopic endometrium, while ERα levels were comparable (Figure 1C,D).

### 3.2. ESCs from Different Lesion Types Show Distinct Proliferation and Migration Phenotypes

**Figure 2:**
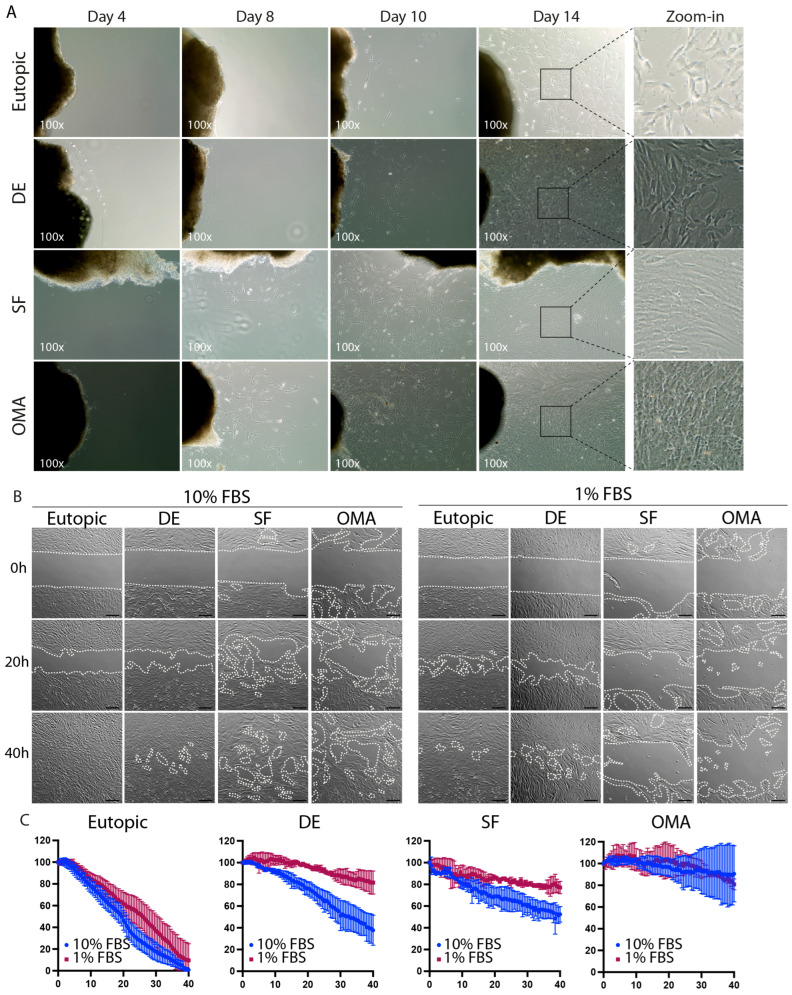
Distinct proliferative and migratory behavior in ESCs from different lesion types. (**A**) ESCs were left to grow out from tissue pieces from the indicated lesion types and images were taken after 4, 10, and 14 days. (**B**) The proliferative and migratory capabilities of ESCs were assessed via a gap closure assay in medium supplemented with standard (**left**) or low (**right**) concentration of FBS. The relative size of the gap (normalized to the initial gap size) is indicated in the top left corner of the images. Dotted lines mark the cell front borders. (**C**) The graphs show the relative gap size normalized to the initial gap size. Blue: 10% FBS; red: 1% FBS. Data is presented as mean +/− SD. Scale bars represent 200 µm for (**B**). (**A**) n = 5 for endometrioma, n = 6 for all other groups. (**B**,**C**) n = 4 for endometrioma; n = 5 for all other groups. DE: deep endometriosis, SF: superficial endometriosis, OMA: endometrioma.

When growing out ESCs from different lesions, we found interesting lesion-type-specific differences: ESCs from DE lesions behaved similarly to those from eutopic endometrium, with a relatively slow migration out of the tissue (Figure 2A). At day 14, cells from eutopic endometrium and deep lesions showed cell aggregates mainly surrounding the primary tissue with no specific migratory orientation. In contrast, cells that were grown out from endometrioma and superficial lesions presented with much faster migration out of the tissue. In these lesions, sparse cells with an elongated shape rapidly separating from the tissue were observed as early as on day 8 of culture. By day 14, ESCs from endometrioma and superficial lesions showed a dense colony organization all over the culture surface, while eutopic ESCs and ESC from DE lesions remained in patches surrounding the tissue (Figure 2A), indicating differences in cell migration depending on the type of the lesion from which the ESCs were initially derived.

To further characterize the migratory behavior of ESCs from the different lesion types in a standardized manner, we analyzed their ability to cover a gap (Figure 2B,C). Under normal growth conditions (10% FCS, left panel), cells from eutopic endometrium showed a uniform cellular front and were able to cover the gap completely in a period of 40 h. In contrast, cells from endometriotic lesions were unable to cover the gap completely within 40 h, while showing enhanced migratory behavior (Figure 2B,C). Notably, this effect was not uniform among ESCs from different ectopic lesion types and was most pronounced in ESCs obtained from endometrioma (Figure 2B,C).

When ESCs were cultured in low nutrient medium (1%FCS instead of 10%FCS), ESCs from eutopic endometrium shifted from a proliferative to a more migratory phenotype (Figure 2B), leading to dispersed cells across the gap during an initial phase (10–30 h) and an almost completely confluent cell layer by 40 h (Figure 2B). Diminished proliferation was even more pronounced in ESCs from superficial and deep infiltrating lesions, which were only able to reduce the gap size to about 80% of its initial size (Figure 2C,D). ESCs from endometrioma, which were already highly motile and inefficient in covering the gap in normal growth conditions, showed minor effects upon nutrient restriction with similarly low gap covering in low nutrient (1% FCS) or normal nutrient (10% FCS) medium (Figure 2C,D).

### 3.3. Reduced mRNA Expression of Genes Involved in Cell Cycle and Proliferation

**Figure 3:**
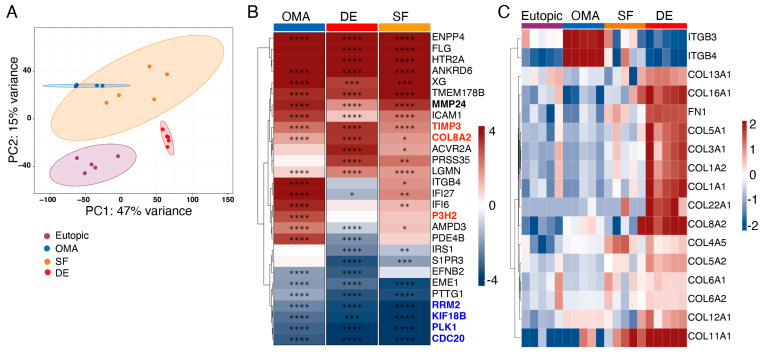
Decreased expression of genes involved in cell proliferation in ESCs from EM lesions. RNA was isolated from ESCs from eutopic tissue or the indicated lesions and subjected to RNA sequencing. (**A**) Principal component analysis plot. (**B**) Top 30 genes that were differentially expressed (log2 fold change > 2) in all three comparisons (*p* < 0.05; DE vs. eutopic (DE), OMA vs. eutopic (OMA), and SF vs. eutopic (SF)), presented as summarized data. Asterisks indicate significant upregulation (red) or downregulation (blue) when corrected for multiple testing (* q < 0.05, ** q < 0.01, *** q < 0.001, **** q < 0.0001; FDR-adjusted *p* values). (**C**) Log2 scaled and normalized counts for the indicated genes; each column represents one sample from an individual patient. Sample size: n = 5 for each group. DE: deep endometriosis, OMA: endometrioma, SF: superficial endometriosis.

To further explore the mechanisms underlying the observed differences in cell proliferation and migration, we performed RNA sequencing on ESCs from the different lesion types. These analyses not only showed that ESCs from eutopic endometrium differed from those from EM lesions but also revealed that ESCs from each lesion type clustered distinctly (Figure 3A). Gene set enrichment analysis revealed that ESCs from all EM lesion types showed altered expression of genes involved in cell division/proliferation (e.g., *CDC20*, *PLK1*, *KIF18B*, *RRM2*), and cell migration/adhesion (e.g., *EFNB2*, *ITGB4*, *ICAM1*; Figure 3B,C), which matches our cell culture observations. These findings were confirmed by qPCR for *CDC20*, *ITGB4*, and *ICAM1* in a separate set of ESCs (Supplementary Figure S3). Expression levels were not uniform in ESCs from the different EM lesions, indicating distinct pathophysiological mechanisms in each lesion type. In addition to genes involved in cell cycle and migration, the expression level of genes involved in the organization or building of the extracellular matrix, such as *P3H2* (involved in collagen production), *COL8A2* (encoding for a collagen subunit), and *FN1* (encoding for fibronectin), were increased (Figure 3B). When analyzing expression levels of genes encoding for collagens more in depth, we observed that ESCs from deep endometrial lesions expressed the highest levels of genes encoding for different collagen subunits, while those from superficial lesions expressed intermediate, and those from endometrioma expressed low levels (Figure 3C. Integrins *ITGB3* and *ITGB4* (encoding for β3 and β4 integrin), on the other hand, were specifically increased in ESC from endometrioma (Figure 3C, Supplementary Figure S3).

### 3.4. Altered Protein Expression in ESCs from EM Lesions

**Figure 4:**
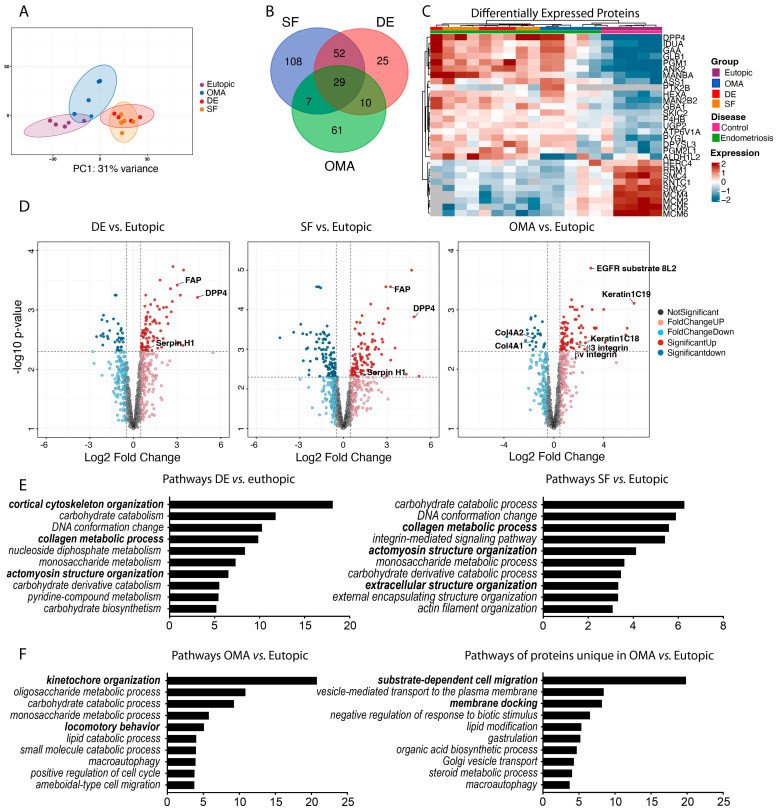
Distinct protein expression profile in ESCs from different EM lesions. Protein was isolated from ESCs from eutopic endometrium (n = 5), superficial (SF, n = 5), or deep (DE, n = 4) endometriotic lesions, or endometrioma (OMA, n = 5), and subjected to proteomics analyses. (**A**) Principal component analysis plot. (**B**) Venn diagram of differentially expressed genes in the comparisons SF vs. eutopic, DE vs. eutopic, and OMA vs. eutopic. (**C**) Heat map for the 29 proteins that were significantly altered in all three comparisons; each column represents one individual. (**D**) Volcano plots for the three comparisons: log2 fold change > 0.5 and q < 0.05 (corrected *p*-values for false discovery rate (FDR)) were considered significantly up- or downregulated. (**E**,**F**) Pathway analyses for significantly altered proteins in the indicated comparisons. The graphs show the top 10 significantly altered pathways, pathways of specific interest are highlighted in bold font. *Y*-axis: enrichment scores. ((**E**), right side) Pathway analysis for the proteins that were uniquely altered in ESCs from endometrioma but not in those from superficial or deep endometriotic lesions. *Y*-axis: enrichment scores.

Similar to the results obtained from RNA expression analyses, proteomics analyses also showed that ESCs from different lesion types clustered in distinct groups (Figure 4A–C) with 29 shared proteins that were altered in all three ESC types when compared to ESCs from eutopic endometrium, 52 proteins shared between ESCs from superficial and deep endometriosis lesions, and 61 proteins uniquely affected in ESCs from endometrioma (Figure 4B). Consistent with the RNASeq results, pathway analysis revealed that proteins involved in collagen metabolic processes, such as Serpin H1, α-N-Acetylglycosaminidase, and DPP4, were significantly enriched in ESCs from deep and superficial endometriosis (Figure 4D,E). Further, fibroblast-activated protein (FAP), a molecule which, together with DPP4, promotes tissue infiltration [39], was significantly elevated. On the other hand, in endometrioma ESCs, collagen 4 levels were reduced, while cytokeratin C18 and C19, both involved in intracellular structure integrity and cell migration [40,41], were enhanced (Figure 4D). Consistent with our mRNA data and the increased migratory phenotype of ESCs from endometrioma, αv and β3 integrins, which are involved in fibrotic processes and cellular tissue infiltration [42], were enhanced in these cells (Figure 4D). Pathway analyses further indicated that in ESCs from endometrioma, proteins involved in locomotory behavior and substrate-dependent cell migration were increased (Figure 4F). Remarkably, pathway analysis showed alterations in several catabolic and metabolic processes in all EM ESCs (Figure 4E,F), which is consistent with a reduced ability to proliferate upon nutrient deprivation. Overall, our proteomics analyses show clear differences between ESCs from eutopic endometrium and the different lesion types, with cells from deep and superficial lesions sharing more similarities to each other than to those isolated from endometrioma.

### 3.5. ESCs from Deep and Superficial Lesions Showed Increased Contractibility

**Figure 5:**
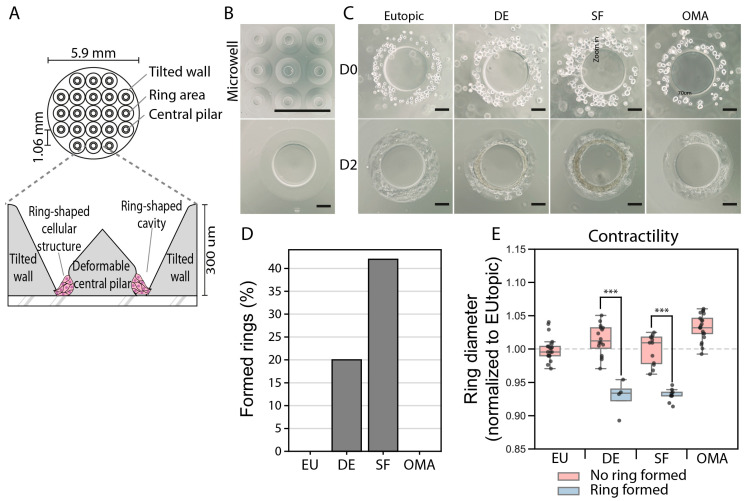
ESCs from deep and superficial lesions show increased contractility. (**A**) Schematic representation of the culture wells used in the contractility assay. (**B**) Bright-field pictures of the empty microwells. (**C**) Representative pictures of microwells 1 and 2 days after seeding with ESCs from the indicated lesions. (**D**) Percentage of wells that formed rings. (**E**) Relative diameter of the ring, normalized to the diameter of the wells seeded with ESCs from eutopic tissue. Each dot represents one well; for each lesion n = 4 patient samples were used; and the measurements were performed in triplicate. EU: eutopic, DE: deep endometriosis, SF: superficial endometriosis, OMA: endometrioma. *** *p* < 0.001, Student’s *t* test. Scale bars: 1.5 mm (top panel in (**B**)) or 20 mm (all other images).

Given the changes in genes involved in extracellular matrix organization, we assessed whether ESCs from different lesion types show differences in their connectivity and contractibility—key functions of fibroblasts in wound healing but also accounting for clinical features in EM, such as pain sensation and infertility [43,44]. For this aim, we employed a specialized cell culture system that allows determining the ability of cells to connect with each other and to contract (Figure 5). This system showed that ESCs from superficial and deep endometriosis lesions, but not ESCs from eutopic endometrium or endometrioma, were able to form rings around a central pillar-like structure (Figure 5C) and to contract the diameter of those rings, indicating that they not only show superior connectivity but also enhanced contractibility when compared to cells from eutopic endometrium or from endometrioma (Figure 5C–E). Overall, these findings suggest that the observed distinct alterations in mRNA and protein expression levels resulted in clear functional differences among ESCs from different lesion types.

## 4. Discussion

Here, we demonstrate that ESCs isolated from different EM lesions show lesion-type specific alterations in migration, nutrient use, and production of extracellular matrix. The complexity and heterogeneity of EM has long been neglected, and robust, representative in vitro models of affected cells from different lesion types are sparse, although such systems would be a potent tool to unravel disease mechanisms and to test novel treatment options [22,45]. In our study, we utilized a migration-based method to isolate and expand ESCs, which was—in contrast to enzymatic digestion—successful and better suited for the isolation of pure ESCs from EM lesions. Our results show that ESCs from endometriotic lesions presented with an increased migratory potential were inferior in closing a gap, and had an altered expression of several genes and proteins involved in cell migration/motility, production of the extracellular matrix, and cellular metabolism. In general, ESCs from different lesions mirrored aspects of the lesion type from which they were initially obtained (e.g., high levels of pro-fibrotic factors in ESCs from deep and to some extent in superficial, but not in those from endometrioma; an overview is given in Supplementary Table S2). This suggests that ESCs isolated via migration are an excellent model to study endometriosis heterogeneity and to develop/test personalized treatment options.

In line with previous reports, levels of the fibroblast marker CD90 were reduced in ESCs from deep endometriosis lesions [46]. Fibroblasts expressing high levels of CD90 produce IL-6, IL-8, and MCP-1 [47], suggesting that these cells are linked to inflammatory processes of the disease. The reduced number of CD90^high^ cells in ESCs from deep EM lesions, but not in ESCs from other lesion types, indicates lesion-specific differences in pro-fibrotic vs. inflammatory processes. However, to understand the physiological relevance of these findings, further studies (e.g., co-culture experiments with immune cells) are required.

Recently, an integrated single cell reference atlas of human endometrium has been published, where data from 63 patients and over 300,000 nuclei from endometrium had been sequenced [48]. This study sheds light into the expression profiles of healthy endometrium and alterations in endometriosis patients; however, most samples included in this atlas were obtained from eutopic endometrium, and the disease heterogeneity of endometriosis has not (yet) been assessed. Thus, it will be of great interest to integrate our observations from ESCs of different endometrial lesions with those in the single cell reference atlas.

The lesion-type-specific differences of ESCs regarding their migratory behavior during isolating and the gap closure assays were remarkable. These findings show that ESCs from EM lesions are inferior in covering a gap when compared to ESCs from eutopic endometrium, they are highly dependent on nutrient availability, their migratory and proliferative potential is altered, and these differences are distinct in ESCs from different lesion types. In specific, ESCs from endometrioma—and to a lower extent also those from superficial EM—showed a pronounced migratory behavior, while failing to efficiently close a gap. ESCs from deep EM, on the other hand, showed increased proliferation, and in that aspect resembled the behavior of eutopic endometrium. These differences in migration and proliferation reflect clinical features of different lesions and could explain individual differences in invasiveness and lesion progression. ESC migration is influenced by the interaction with the surrounding tissue and extracellular matrix proteins. In fibrosis, collagen and fibronectin, which are deposited upon repeated injury/repair cycles, provide a scaffold for fibroblasts and thereby promote the invasive phenotype of ESCs [49,50]. Thus, the observed lesion-specific differences in the expression of genes and proteins involved in collagen metabolism and in the reorganization of the extracellular matrix are of particular interest as they might correlate with scar formation and could potentially predict the progression of a lesion. Overall, the unique characteristics of ESCs from different endometriotic lesions hold the potential to understand—and therapeutically target—the mechanisms underlying their invasiveness and their potential to promote EM in a personalized, lesion-specific manner.

The observed impact of nutrient restriction on ESCs’ migration and proliferation, particularly in cells from superficial lesions, paired with alterations in proteins involved in cellular metabolic and catabolic processes, underscores the high metabolic demand for ESC expansion. This is well in line with postulated similarities of ESCs with cancer cells, including resemblance to the Warburg effect [51,52] and aberrant glycolysis [53,54]. Thus, at least for certain disease manifestations, targeting the cellular metabolism might potentially offer novel, nonhormonal treatment options [55]. In addition, these findings align well with metabolic studies that show altered glycolysis, TCA intermediates, and lipid metabolism in endometriosis patients [56], and link the described changes in the metabolic profile in patient’s peritoneal fluid and serum with alterations in the behavior and metabolic need of ESCs.

The elevated expression of ERβ in ectopic lesions is also of interest, as increased ERβ expression had previously been linked to immune evasion of ESCs via upregulation of CD47 [13,57,58], highlighting the interplay between hormone receptors and immune modulation in EM. Additionally, ERβ is considered a key molecule in repeated tissue damage and repair mechanisms [59,60,61], it influences cell motility via focal adhesion kinase activation [62], and it promotes lesion growth and immune dysregulation [63]. On the other hand, it has been shown that ERβ can mitigate fibrosis development [64], although in the endometrium, fibrosis development was associated with diminished ERα levels [65]. The heightened estrogen production in EM, coupled with endometriotic ESCs expressing markedly elevated ERβ levels compared to eutopic ESCs [13,66], underscores the importance of a tight regulation of ER signaling. The observed differences in ERβ expression in ESCs from different EM lesions highlight the need to evaluate its role in lesion progression for each patient and each lesion individually. Such differences in ER expression might also link to the clinical observation that not all EM patients respond equally to hormone treatment with one-fourth to one-third of patients not responding to first-line hormone therapy [67]. Thus, evaluation of ERα/β expression levels on ESCs holds the potential to help guiding decisions on the use of hormone-based treatment options. Of note, ER expression was observed in ESCs from EM lesions without adding any 17-β estradiol (E2) to the culture medium, validating ESC cultures as a model for the in vitro study of estrogen receptor regulation.

Finally, the increased contractility of ESCs from superficial and deep endometriosis lesions is of high relevance as increased fibroblast contractility is associated with tissue scarring, compromised organ function, and pain sensation [43,44], linking our findings to important clinical features of EM, including pain and infertility. Under physiological circumstances, fibroblasts become activated upon tissue injury and start to secrete higher amounts of extracellular matrix proteins [68]. Coupled with increased contractility, these cells support wound closure and healing, upon which they regain an inactive state [68]. In fibrotic diseases, including EM, the return to quiescence is disturbed, and persistently activated fibroblasts promote the formation of scar tissue that ultimately compromises organ function [44]. In this regard, the high expression of αvβ3 integrin is of particular interest, as this integrin promotes fibroblast contraction and fiber stiffness, while suppressing the return to quiescence [69]. Activated fibroblasts also secrete pro-algesic factors, such as nerve growth factor (NGF), IL-6, and IL-1β [43]. These molecules do not only promote pain sensation, but can also enhance nerve growth and the attraction of immune cells, which further aggravates tissue scarring and pain perception. Notably, the upregulation of factors associated with a hyperactivated fibroblast state were more pronounced in ESCs from superficial and deep lesions, which is in line with the clinical observation that patients with endometrioma do typically not show pain while those with superficial and deep lesions do.

While our results show that ESCs from different EM lesion types are a valuable model to study EM heterogeneity as they retain key features of different lesion types, a major limitation of our study is the absence of endometrial tissue from healthy controls without endometriosis, as obtaining such samples requires invasive procedures in healthy women. In future studies, it would be of great interest to include ESCs from completely healthy women to explore the molecular differences between endometriotic lesions, eutopic ESCs that might be influenced by the presence of EM lesions, and eutopic cells from completely unaffected, healthy women. In addition, it should be taken into consideration that in vitro cultures, as those used in ours, can alter cell behavior, and the microenvironment that is present in endometriotic lesions is not fully recapitulated in our in vitro setting (e.g., absence of other cell types such as epithelial cells or immune cells).

## 5. Conclusions

Our study demonstrates significant differences between ESCs from different types of EM lesions regarding proliferation, migration, extracellular matrix composition, and contractility, which highlights the importance to utilize lesion-specific models to investigate and fully understand EM and its heterogeneity. We also demonstrate that EM ESCs have an increased nutrient demand and respond to nutrient deprivation with increased motility. On the other hand, they secrete factors that promote tissue scarring and pain sensation, contributing to clinical features of EM. Overall, our findings help to better understand the complexity of EM, which will ultimately help to develop more effective diagnostic tools and targeted treatment options.

## Data Availability

The original data presented in the study are openly available: Proteomics data have been deposited in the PRIDE repository (PXD066685); RNA sequencing data have been deposited in NCBI’s gene expression omnibus (GEO; GSE303635). All other data are available in the manuscript or the accompanying Supplementary Files.

## Supplementary Materials

The following supporting information can be downloaded at: https://www.mdpi.com/article/10.3390/cells14231891/s1.

## Abbreviations

The following abbreviations are used in this manuscript:

DE	Deep Endometriosis
EEC	Endometrial Epithelial Cell
EM	Endometriosis
EpCAM	Epithelial Cellular Adhesion Molecule
ER	Estrogen Receptor
ESC	Endometrial Stromal Cell
FGCZ	Functional Genomics Center Zürich
OMA	Endometrioma
PBS	Phosphate-Buffered Saline
SF	Superficial
