# Supplementary material for: Endometrial Stromal Cells from Endometriosis Patients Reflect Lesion-Type-Specific Heterogeneity
Source: Cells. 2025 Nov 28;14(23):1891. doi: 10.3390/cells14231891 (PMC12691305; doi:10.3390/cells14231891)
Supplement: Supplementary file 1 [file cells-14-01891-s001.zip › cells-3950509-supplementary.pdf]

Supplementary Material to
Stromal Cells from Endometriosis Patients Reflect Lesion Type-Specific Heterogeneity

Authors: Daniel Rodriguez Gutierrez[1]*, Marianne R. Spalinger[1]*, Alina Astourian[1], Olivera Evrova[1], Lucie Berclaz[1], Monique Hartmann[1], Ioannis Dedes[2], Patrick Imesch[2], Julian M. Metzler[2], Isabelle Witzel[2], Mohaned Shilaih[1], Valentina Vongrad[1], Brigitte Leeners[1]

[1] Department of Reproductive Endocrinology, University Hospital Zurich, Zurich, Switzerland

[2] Department of Gynecology, University Hospital Zurich, Zurich, Switzerland

*Equal contribution

Supplementary Methods

Antibodies used for Flow Cytometry. Surface staining: APC-Cy7-labeleld anti-CD90 (BioLegend, #328132 1:100), APC-labeled anti-CD10 (BioLegend, #312210, 1:150), AlexaFluor-labelled anti-EpCAM (BioLegend, #324209, 1:100). Intracellular staining: AlexaFluor488 labelled anti human ERα (Abcam, #ab-194150, 1:100) and PE-labelled anti-human ERβ (Abcam, #AB205541-1001, 1:200).

Antibodies used for Immunofluorescence staining. The following primary antibodies were used: rabbit anti human EpCAM (Epithelial cellular adhesion molecule; abcam, #ab223582, 1:500), rabbit anti-human-CD90 (Sigma #HPA003733, 1:200), rabbit anti-human CD10 (Sigma # SAB4300659, 1:200), mouse anti-human vimentin (VIM, Invitrogen, MA5-11883, 1:200), and mouse anti-human α smooth muscle actin (α-SMA, Sigma #A2547, 1:500). The following secondary antibodies were used: AlexaFluor 555 labelled goat anti-mouse, Thermo Fisher, #A34570; AlexFluor488-labelled goat anti-rabbit, Sigma-Aldrich, #A11008. All secondary antibodies were diluted 1:500.

Contractility Assay. ESCs (P3) were washed with PBS, detached with trypsin, resuspended in ESC culture media, passed through a 70 um cell strainer. 1x106 cells were seeded per well in a 24 well 4Dcell SmartHeart plate. Images were taken at the time of seeding, after 24 h, and after 48 h, to assess the ring formation. Contractility was analyzed by measuring the area of the 6kPa polyacrylamide gel pillar after 48 h.

Proteomics sample preparation. BB Protein concentration was determined using the Lunatic UV/Vis polychromatic spectrophotometer (Unchained Labs). 20ug protein per sample in final 4%SDS were boiled at 95°C for 10 minutes followed by 1 minute high-intensity focused ultrasound (HIFU). Proteins were reduced with 5 mM TCEP(tris(2-carboxyethyl)phosphine) and alkylated with 15 mM chloroacetamide at 30°C for 30 min. Samples were processed using the single-pot solid-phase enhanced sample preparation (SP3). The SP3 protein purification, digest and peptide clean-up was performed using a KingFisher Flex System (Thermo Fisher Scientific) and Carboxylate-Modified Magnetic Particles (GE Life Sciences; GE65152105050250, GE45152105050250) [1, 2]. Beads were conditioned following the manufacturer's instructions, consisting of 3 washes with water at a concentration of 1 µg/µl. Samples were diluted with 100% ethanol to a final concentration of 60% ethanol. The beads, wash solutions and samples were loaded into 96 deep well- or micro-plates and transferred to the KingFisher. Following steps were carried out on the robot: collection of beads from the last wash, protein binding to beads, washing of beads in wash solutions 1-3 (80% ethanol), protein digestion (overnight at 37°C with a trypsin:protein ratio of 1:50 in 50 mM Triethylammoniumbicarbonat (TEAB)) and peptide elution from the magnetic beads using MilliQ water. The digest solution and water elution

were combined and dried to completeness and re-solubilized in 20 μL of MS sample buffer (3% acetonitrile, 0.1% formic acid).

Mass Spectrometry. Mass spectrometry analysis was performed using an Orbitrap Exploris 480 mass spectrometer (Thermo Fisher Scientific), equipped with an EASY ion source (Thermo Fisher Scientific) and coupled to an Evosep One (EvoSep Biosystems). The samples were separated using the Whisper100 Zoom 20 SPD method on an Aurora Elite column (IonOpticks, 15 cm, 75 μm ID, 1.7 μm C18), heated at 50 °C. Solvent A corresponded to 0.1% formic acid and solvent B to 0.1% formic acid, 99.9% acetonitrile. Peptides were loaded onto an Evotip (EvoSep Biosystems) according to the manufacturer's instructions. For the analysis of the individual samples, the mass spectrometer was operated in data-independent mode (DIA). DIA scans covered a range from 400 to 960 m/z in windows of 8 m/z. The resolution of the DIA windows was set to 30'000, with a normalized AGC target value of 1'000%, the maximum injection time set to auto and a fixed normalized collision energy (NCE) of 30 %. Each instrument cycle was completed by a full MS scan monitoring 396 to 1'000 m/z at a resolution of 60'000. The mass spectrometry proteomics data were handled using the local laboratory information management system (LIMS)[3] and all relevant data have been deposited to the ProteomeXchange Consortium via the PRIDE (http://www.ebi.ac.uk/pride) partner repository with the data set identifier PXD066685.

Spectral library generation and protein quantification. The identification and quantification of proteins from MS data was performed using the DIA-NN workflow (version 1.9.1, [4]). The following parameters for the library-free search were used: precursor charge +2 and +3, precursor mass range 400 m/z to 1500 m/z, fragment mass range 200 m/z to 1800 m/z, mass accuracy MS1 15 ppm, MS2 20 ppm, enzyme specificity trypsin/P allowing one missed cleavage, fixed modification carbamidomethylation of cysteine. The in-silico spectra were generated using a canonical protein database for homo sapiens, concatenated with common protein contaminants (Taxonomy 9606, UP000005640 reference proteome with one protein sequence per gene, release date 20230330) and the maximum false discovery rate (FDR) was set to 0.01.

Statistical Modelling of proteomics data. The R package prolfqua [5] was used to analyze the differential expression and to determine group differences, confidence intervals, and false discovery rates for all quantifiable proteins. Starting with the report.tsv file generated by DIANN, which does report the precursor ion abundances for each raw file, we determined protein abundances by first aggregating the precursor abundances to peptidoform abundances. Then, we employed the Tukeys-Median Polish to estimate protein abundances. Furthermore, before fitting the linear models, we transformed the protein abundances using the variance stabilizing normalization [6].

RNA sequencing. RNA concentration and integrity was determined with a Qubit® Fluorometer and an Agilent Fragment Analyzer. Samples with a 260 nm/280 nm ratio between 1.8–2.1 and a 28S/18S ratio within 1.5–2 were further processed with the Universal Plus mRNA-Seq Library Preparation Kit (Tecan Life Sciences, AG, Switzerland). RNA samples were polyA enriched; reverse-transcribed; fragmented, end-repaired and adenylated; adapters with unique dual indices (UDI) ligated; fragments containing adapters on both ends selectively enriched with PCR; and libraries sequenced on an Illumina NovaSeq X Plus.

RNA sequencing data analysis. Individual library size ranged from 35-48 million reads and analysis was performed using the SUSHI framework [7]: Read quality inspection with FastQC, sequencing adaptor removal with fastp; Pseudo-alignment and transcriptomic counts of the RNA-Seq reads using the Kallisto Bioncoductor R package [8] with the GENCODE human genome build GRCh38.p13 (Release 42) as reference (annotation downloaded on May 2023); Differential expression using the generalised linear model as implemented by the DESeq2 Bioconductor R package; Gene Ontology (GO) term pathway analysis using both the hypergeometric over-representation test via the 'enricher' function; and gene-set enrichment analysis via the 'GSEA' function, of the clusterProfiler Bioconductor R package. All R functions were executed on R version 4.5 (R Core Team, 2020) and Bioconductor version 3.20. Figures were generated using the exploreDE Shiny application [9] using the data generated from the above steps.

Supplementary References

1. Hughes, C.S., et al., *Ultrasensitive proteome analysis using paramagnetic bead technology.* Mol Syst Biol, 2014. **10**(10): p. 757.
2. Leutert, M., et al., *R2-P2 rapid-robotic phosphoproteomics enables multidimensional cell signaling studies.* Mol Syst Biol, 2019. **15**(12): p. e9021.
3. T\ rker, C., et al., *B-Fabric: the Swiss Army Knife for life sciences*, in *Proceedings of the 13th International Conference on Extending Database Technology*. 2010, Association for Computing Machinery. p. 717–720 , numpages = 4.
4. Demichev, V., et al., *DIA-NN: neural networks and interference correction enable deep proteome coverage in high throughput.* Nat Methods, 2020. **17**(1): p. 41-44.
5. Wolski, W.E., et al., *prolfqua: A Comprehensive R-Package for Proteomics Differential Expression Analysis.* J Proteome Res, 2023. **22**(4): p. 1092-1104.
6. Huber, W., et al., *Variance stabilization applied to microarray data calibration and to the quantification of differential expression.* Bioinformatics, 2002. **18 Suppl 1**: p. S96-104.
7. Hatakeyama, M., et al., *SUSHI: an exquisite recipe for fully documented, reproducible and reusable NGS data analysis.* BMC Bioinformatics, 2016. **17**(1): p. 228.
8. Bray, N.L., et al., *Near-optimal probabilistic RNA-seq quantification.* Nat Biotechnol, 2016. **34**(5): p. 525-7.
9. Peter Leary, H.R., *exploreDE Interactive Shiny App.* Zenodo, 2024.

Supplementary Figures

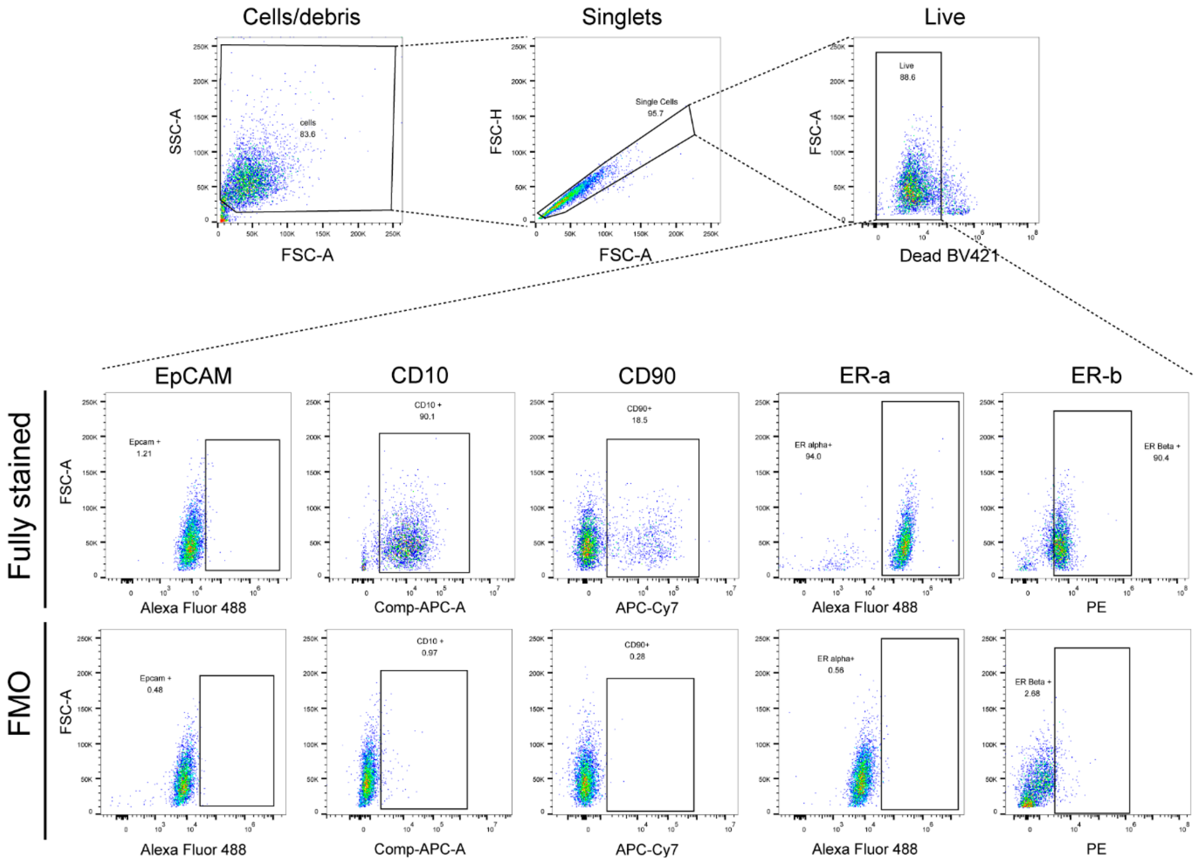

Supplementary Figure S1. Flow cytometry gating strategy. Representative dot plots from flow cytometry analyses. Cell debris, doublets, and dead cells were excluded prior to assessing positive and negative populations for the indicated markers. FMO (Fluorescence-minus-one): control samples that were fully stained except for the one color that is shown on the x-axis of the respective plots. FMO controls were used to set the boundary between negative and positive cells.

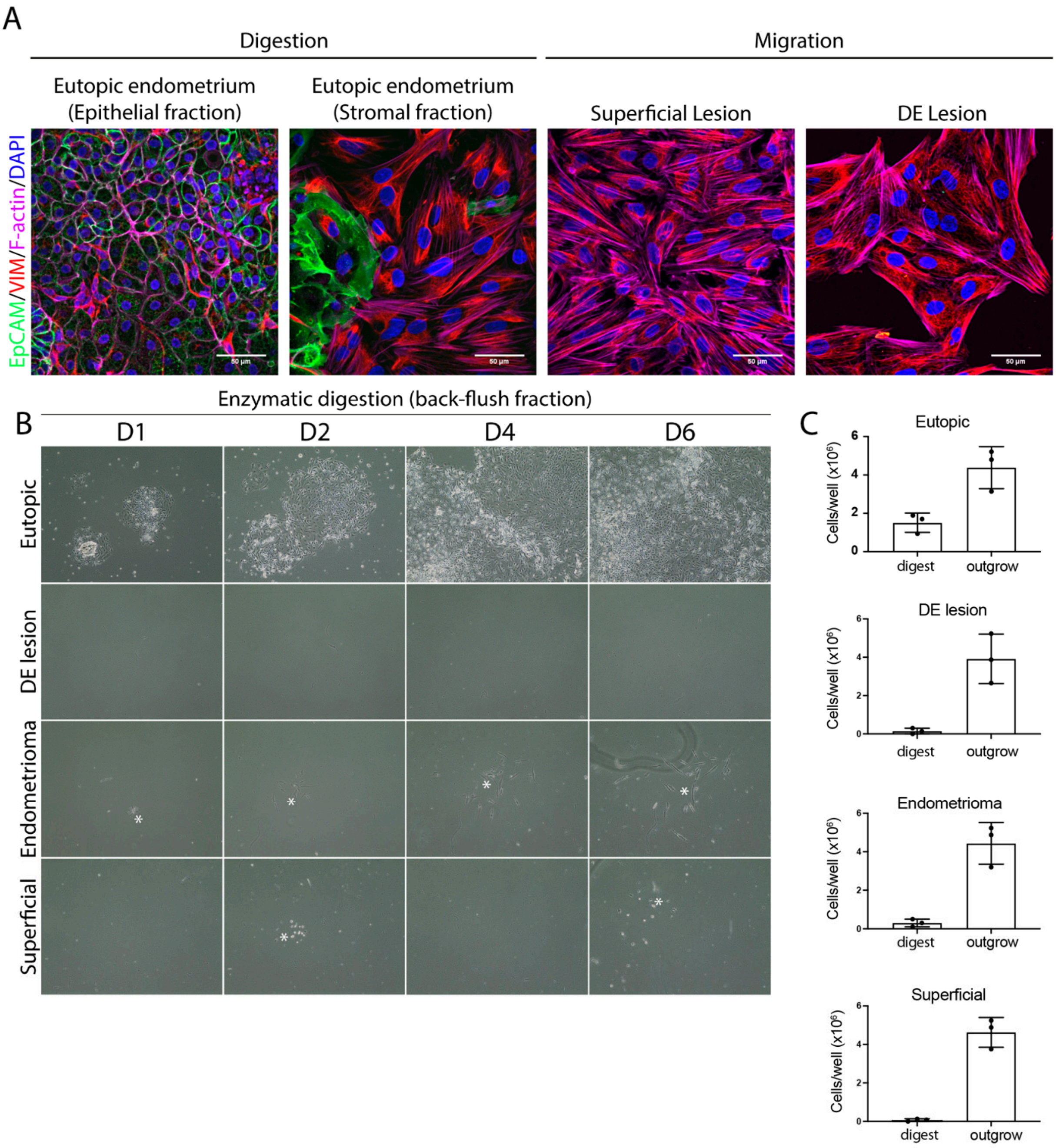

Supplementary Figure S2. Comparison between enzymatic digestion and migration-based ESC isolation. A) Left side (Digestion): Tissue samples from the indicated endometriosis lesion types were digested in medium containing 1 mg/ml collagenase I, filtered through a 70 um nylon mesh. The flow-through (epithelial fraction) and the cells that were retained in the filter (stromal fraction) were stained for the indicated markers and assessed under a fluorescence microscope. Right side (Migration): Tissue samples were placed on cell culture dishes and cells left to outgrow/migrate out from the tissue for up to 20 days prior to staining for the indicated markers and assessment under a fluorescence microscope. B) Tissue samples were placed on cell culture dishes and cells left to outgrow/migrate out from the tissue for 6 days. Pictures were taken after 1, 2, 4 and 6 days. DE: Deep endometriosis. C) Number of cells that were obtained 14 days after isolation with the digestion method or the outgrowing method. n=3 for each lesion type in each of the analyses.

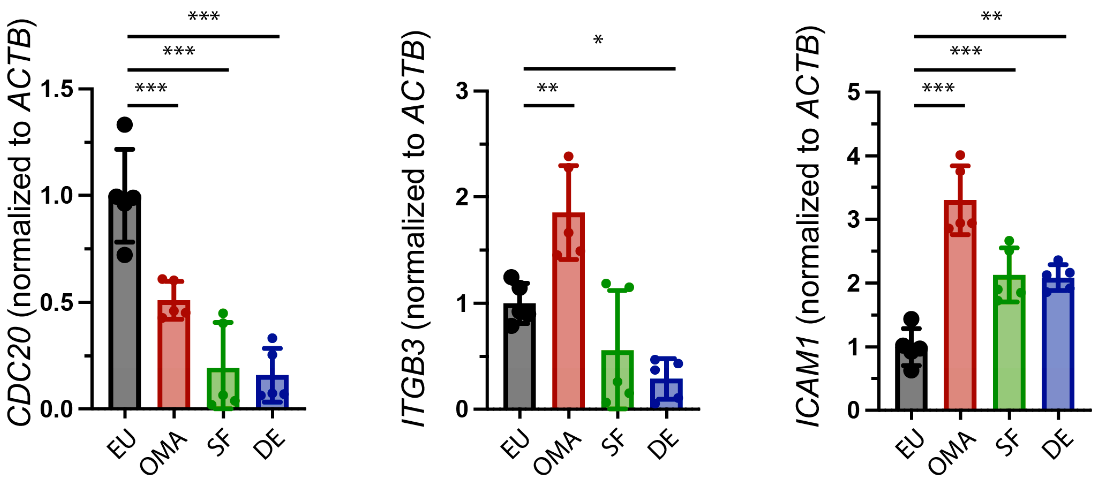

Supplementary Figure S3. Quantitative PCR in ESC from different lesion types. RNA was extracted from ESCs and analyzed for mRNA expression of the indicated genes. Data is normalized to the house-keeping gene *ACTB* and the mean of ESC from eutopic endometrium. EU: eutopic, OMA: endometrioma, SF: superficial endometriosis, DE: deep endometriosis. Asterisks indicate significant differences (*p<0.05, **p<0.01, ***p<0.001), n=5 for each lesion type.

Supplementary Table S1. Patient Characteristics.

Patient	Type of biopsy	Age	Cycle day	Hormonal Treatment	Pain medication	Shown in Figures*
1	eutopic	43	12	NO	NSAID	1A+B, 2A, 5
2	eutopic, DE	32	13	NO	NSAID	1A-D, 2A-C, 3, 4, 5
3	eutipoc, DE	40	21	NO	NSAID	1A-D, 2A-C, 3, 4
4	eutopic, SF	29	3	NO	NSAID	1A-D, 2A-C, 3, 4, 5
5	eutopic, SF	37	1	NO	NSAID	1A-D, 2A-C, 3, 4
6	eutopic	34	3	NO	NSAID	1A+B,
7	eutopic, OMA	33	12	Visanne	NSAID	1A-D, 2A-C, 3,4, 5
8	SF	44	10	Visanne	NSAID	1A-D, 2A-C, 3, 4, 5
9	SF	41	10	NO	NSAID	1A-D, 2A-C, 3, 4
10	SF	28	12	Visanne	NSAID	1A-D, 2A-C, 3, 4, 5
11	SF	31	24	NO	NSAID	1A+B, 2A, 5
12	DE	31	10	Cerazette	NSAID	1A-D, 2A-C, 3, 4
13	DE	26	14	NO	NSAID	1A-D, 2A-C, 3, 4
14	DE	33	8	Dienogest	NSAID	1A-D, 2A-C, 3, 5
15	DE	33	2	Visanne	NSAID	1A+B, 2A-C, 5
16	DE	43	12	Visanne	NSAID	1A+B, 2A, 5
17	OMA	40	4	NO	NSAID	1A-D, 2A-C, 3, 4, 5
18	OMA	36	24	NO	NSAID	1A-D, 2A-C, 3, 4, 5
19	OMA	42	3	NO	NSAID	1A-D, 2A-C, 3, 4
20	OMA	42	11	NO	NSAID	1A-D, 2A, 3, 4, 5

*Analyses performed with ESC from this patient are shown in these figures.

Supplementary Table S2. Clinical characteristics compared to observations in ESC cultures.

	Clinical presentation	observations in in cell culture
**Endometrioma**	*often asymptomatic, low pain level*	*low proliferation, low contractility*
	Dissemination to other sites common	*highly migratory, integrins that promote dissemination up, moderate expression of integrins that mediate infiltration*
	less painful than other types	*contractility low*
		high metabolic demand
**Superficial**	*Mild symptoms*	*intermediate phenotypes*
	Moderate invasiveness	*intermediate migration, FAP/DPP4 up (promote tissue infiltration)*
	painful	*Collagen and pro-fibrotic factors up, high contractility*
	irregular cycle	*High expression of pro-fibrotic factors, contractility high*
**Deep Infiltrating Endometriosis**	*Highly fibrotic*	*high expression of pro-fibrotic factors*
	Tissue invasion	*Migration of cells moderate, FAP/DPP4 high (promote tissue infiltration)*
	High pain level	*high contractility*
